# Heart rate and breathing effects on attention and memory (HeartBEAM): study protocol for a randomized controlled trial in older adults

**DOI:** 10.1186/s13063-024-07943-y

**Published:** 2024-03-15

**Authors:** Kaoru Nashiro, Hyun Joo Yoo, Christine Cho, Andy Jeesu Kim, Padideh Nasseri, Jungwon Min, Martin J. Dahl, Noah Mercer, Jeiran Choupan, Paul Choi, Hye Rynn J. Lee, David Choi, Kalekirstos Alemu, Alexandra Ycaza Herrera, Nicole F. Ng, Julian F. Thayer, Mara Mather

**Affiliations:** 1 University of Southern California, Los Angeles, USA; 2 Center for Lifespan Psychology, Max Planck Institute for Human Development, Berlin, Germany; 3 NeuroScope Inc., New York, USA; 4 Lumos Labs, Inc., San Francisco, USA; 5 University of California, Irvine, USA

**Keywords:** Heart rate oscillations, Heart rate variability biofeedback, Slow-paced breathing, Alzheimer’s disease, Amyloid beta, Perivascular space, Cognition, Memory, Cognitive training, Brain games

## Abstract

**Background:**

In healthy people, the “fight-or-flight” sympathetic system is counterbalanced by the “rest-and-digest” parasympathetic system. As we grow older, the parasympathetic system declines as the sympathetic system becomes hyperactive. In our prior *heart rate variability biofeedback and emotion regulation* (HRV-ER) clinical trial, we found that increasing parasympathetic activity through daily practice of slow-paced breathing significantly decreased plasma amyloid-β (Aβ) in healthy younger and older adults. In healthy adults, higher plasma Aβ is associated with greater risk of Alzheimer’s disease (AD). Our primary goal of this trial is to reproduce and extend our initial findings regarding effects of slow-paced breathing on Aβ. Our secondary objectives are to examine the effects of daily slow-paced breathing on brain structure and the rate of learning.

**Methods:**

Adults aged 50–70 have been randomized to practice one of two breathing protocols twice daily for 9 weeks: (1) “slow-paced breathing condition” involving daily cognitive training followed by slow-paced breathing designed to maximize heart rate oscillations or (2) “random-paced breathing condition” involving daily cognitive training followed by random-paced breathing to avoid increasing heart rate oscillations. The primary outcomes are plasma Aβ40 and Aβ42 levels and plasma Aβ42/40 ratio. The secondary outcomes are brain perivascular space volume, hippocampal volume, and learning rates measured by cognitive training performance. Other pre-registered outcomes include plasma pTau-181/tTau ratio and urine Aβ42. Recruitment began in January 2023. Interventions are ongoing and will be completed by the end of 2023.

**Discussion:**

Our HRV-ER trial was groundbreaking in demonstrating that a behavioral intervention can reduce plasma Aβ levels relative to a randomized control group. We aim to reproduce these findings while testing effects on brain clearance pathways and cognition.

**Trial registration:**

ClinicalTrials.gov NCT05602220. Registered on January 12, 2023.

**Supplementary Information:**

The online version contains supplementary material available at 10.1186/s13063-024-07943-y.

## Administrative information

Note: the numbers in curly brackets in this protocol refer to SPIRIT checklist item numbers. The order of the items has been modified to group similar items (see http://www.equator-network.org/reporting-guidelines/spirit-2013-statement-defining-standard-protocol-items-for-clinical-trials/).

**Table:** 

Title {1}	Heart rate and breathing effects on attention and memory (HeartBEAM): study protocol for a randomized controlled trial
Trial registration {2a and 2b}.	NCT05602220 [ClinicalTrials.gov] [registered on 12-01-2023]
Protocol version {3}	UP-21-00357; IRB protocol version 11, approved on 15 May 2023.
Funding {4}	This study was supported by USC Epstein Breakthrough Alzheimer’s Research Fund (PI Mather).
Author details {5a}	K. Nashiro: University of Southern California H. Yoo: University of Southern California C. Cho: University of Southern California A.J. Kim: University of Southern California P. Nasseri: University of Southern California J. Min: University of Southern California M.J. Dahl: University of Southern California, Center for Lifespan Psychology, Max Planck Institute for Human Development N. Mercer: University of Southern California J. Choupan: University of Southern California, NeuroScope Inc. P. Choi: University of Southern California H.J. Lee: University of Southern California D. Choi: University of Southern California K. Alemu: University of Southern California A.Y. Herrera: University of Southern California N.F. Ng: Lumos Labs, Inc. J.F. Thayer: University of California, Irvine M. Mather: University of Southern California
Name and contact information for the trial sponsor {5b}	Investigator initiated clinical trial; M. Mather (Principal Investigator) mara.mather@usc.edu
Role of sponsor {5c}	This is an investigator-initiated clinical trial. Thus, the funders did not play any role in the design of the study and collection, analysis, and interpretation of data and in writing the manuscript.

## Introduction

### Background and rationale {6a}

Getting older is the most influential risk factor for Alzheimer’s disease (AD), a progressive neurodegenerative disorder that affects millions of people worldwide. From age 65 on, the risk of AD doubles every 5 years. Understanding why the risk of AD accelerates with age is important for improving our understanding of the disease and developing effective preventive and therapeutic strategies [1]. Previous research has focused on various factors associated with aging, including oxidative stress [2], cardiovascular disease-related factors [3], inflammation [4], and microglial activation states [5]. However, there has been less attention on the potential Alzheimer’s-accelerating role of age-related shifts in the autonomic nervous system.

The parasympathetic and sympathetic branches of the autonomic nervous system show dramatic opposing changes in aging. The primary measure of the parasympathetic system is vagal heart rate variability (e.g., heart rate variability reflecting the acceleration of heart rate during inhalation and the deceleration of heart rate during exhalation), and in a sample of over 8 million people, heart rate variability was estimated to be 80% lower at age 60 than at age 20 [6].

Because sympathetic nerves (and not parasympathetic nerves) release noradrenaline, plasma noradrenaline provides one measure of sympathetic activity. Along with other sympathetic indicators such as sympathetic nerve activity, noradrenaline levels increase in aging, especially during sleep which is normally a time of higher parasympathetic and lower sympathetic activity. For instance, plasma noradrenaline levels were 75% higher during sleep in older men (age 62–80) than in younger men (age 21–28; this study only included males) [7].

Aging affects the noradrenergic system not just in the periphery but also in the brain [8]. In postmortem staging of AD, the earliest stage has been identified as abnormally phosphorylated tau in the locus coeruleus (LC), the brain’s primary source of noradrenaline [9]. In preclinical and early AD, LC damage is likely to lead to increased tonic LC activity, which contributes to the hyperactive noradrenergic activity associated with aging [10–12]. Animal research indicates that manipulations of noradrenergic activity affect the production and clearance of amyloid-β (Aβ) peptides and tau proteins [10]. Thus, changes in the LC are likely to affect noradrenergic activity, which in turn influences the production and clearance of the amyloid peptides and tau proteins involved in the Alzheimer’s disease pathological progression [10].

Genetic and molecular evidence suggests that accumulation of Aβ triggers neuropathological processes leading to cognitive and brain degeneration. Healthy adults with genetic risk of AD tend to have higher plasma Aβ40 and Aβ42 levels [13, 14]. In addition, healthy adults with higher plasma Aβ40 and Aβ42 levels tend to have higher risk of later developing AD [15]. Peripheral levels of Aβ can influence brain Aβ processes, as for instance removing a kidney in a mouse AD model increases plasma Aβ levels as well as brain Aβ deposition [16].

Breathing has a significant impact on the nervous system. In particular, slow-paced breathing has the potential to benefit those suffering from hypoactive parasympathetic (vagal) and hyperactive sympathetic (noradrenergic) activity. In a recent clinical trial examining the effects of slow-paced breathing and heart rate variability biofeedback on emotion-related brain networks (HRV-ER), we included an exploratory outcome measure examining plasma Aβ40 and Aβ42 before and after 4 weeks of the interventions. There were two heart-rate-variability-biofeedback conditions, one using slow-paced breathing to try to maximize heart rate oscillations during practice sessions (Osc+) and the other using personalized strategies to minimize heart rate oscillations during practice sessions (Osc−). Participants in both conditions showed significant changes in plasma biomarkers associated with Alzheimer’s disease [17]. The Osc+ intervention decreased plasma Aβ levels, while the Osc– intervention increased plasma Aβ levels. The current clinical trial aims to reproduce these findings as the primary outcome and to examine several secondary and exploratory outcomes to better understand mechanisms and implications.

### Objectives {7}

Our primary objective for this trial on heart rate and breathing effects on attention and memory (“HeartBEAM”) is *to test whether daily practice sessions involving paced breathing change plasma Aβ levels*, reproducing the initial findings from our HRV-ER clinical trial with slightly modified interventions and a longer intervention period (see Supplementary Table 1 for the differences between the two trials). *Based on our HRV-ER findings, we predict that daily slow-paced breathing will reduce overall plasma Aβ levels (Aβ40 and Aβ42) and increase the plasma Aβ42/Aβ40 ratio.*

We have three secondary objectives. These test whether daily paced breathing will affect the following:

1. *Perivascular space (PVS) volume*: PVS volume estimated using magnetic resonance imaging (MRI) is positively associated with accumulation of Aβ in vessel walls [18, 19]. Consistent with this, MRI-detected perivascular spaces in the centrum semiovale (the large mass of white matter above the corpus callosum) are greater in those with mild cognitive impairment and AD than in healthy controls [20] and are associated with PET amyloid burden [21]. We predict that the intervention will affect PVS volume, reflecting better brain waste clearance. In the current study, we use high-resolution T1w and T2w MRI scans to quantify intervention-induced change in PVS volume in the centrum semiovale [22].
2. *Hippocampal volume*: A rodent AD model indicates that phasic stimulation of the LC can prevent spread of abnormal tau from the LC to cortical memory regions and prevent memory decline [11]. The vagus nerve transmits rhythmic breathing-related signals to the LC, providing phasic stimulation that may enhance noradrenergic and/or dopaminergic inputs to the hippocampus [23]. In our HRV-ER trial, we found that hippocampal subregions with moderate-to-high noradrenergic innervation showed significantly different changes in volume in the Osc+ vs. Osc− conditions, and there was a trend towards an effect on the volume of the whole hippocampus [24]. In the current study, we use high-resolution structural imaging to examine hippocampal volumetric changes.
3. *Learning rates*: To test the effects of daily paced breathing on learning over time, participants play a set of brain training games in addition to completing separate paced breathing sessions every day. We will measure their game performance over time. There are multiple pathways through which the slow-paced breathing intervention might influence learning, including influencing hippocampal function and the availability of Aβ to aggregate as oligomers that interfere with synaptic plasticity.

Other pre-registered outcomes test whether daily paced breathing will affect the following:

1. *Plasma pTau-181/total Tau (tTau)*: We previously found significant differences of the Osc+ and Osc− conditions on the plasma pTau-181/tTau ratio and so will test whether the two-paced breathing conditions affect this ratio differently. Levels of plasma pTau-181 are correlated with CSF pTau-181 [25, 26] and tau PET [27–29], whereas the relationship is weak or non-significant for plasma tTau and CSF tTau [30, 31], suggesting that plasma pTau-181 is more likely to be brain derived than plasma tTau, and changes in how effectively brain waste is cleared to blood may affect plasma pTau-181 levels more than plasma tTau levels. From this perspective, changes in plasma pTau-181/tTau ratio may reflect changes in brain clearance.
2. *Urine Aβ42*: The kidneys help clear Aβ from the body [16]. If paced breathing affects plasma Aβ via its effects on kidney clearance, we should see opposing effects of the breathing intervention on plasma and urine Aβ42 (i.e., reductions of plasma Aβ should be associated with increases in urine Aβ). We will focus on Aβ42 since previous research successfully detected Aβ42 (but not Aβ40) in human urine [16].

We also have some exploratory outcomes. Following up on our secondary outcome of centrum semiovale PVS volume, we will examine regional PVS volume to see if there are differences in PVS change across brain subregions. Likewise, in addition to examining volume of the whole hippocampus, we will examine volume within the LC-targeted ROI including left and right molecular layer, CA3, CA4, and granule cell layer of dentate gyrus, in which we previously found intervention-induced changes [24] and changes in individual hippocampal subregion volumes. Using separate specialized magnetization transfer structural scans, we will examine whether the breathing interventions affect LC MRI contrast [32]. We will test whether there are pre- and post-intervention differences between conditions in cognitive and emotional function, as assessed by various cognitive and emotional assessments listed in Table 4. We also include a pre- and post-intervention electroencephalography (EEG) session for each participant and will test whether there are effects of condition on event-related potentials during an active and passive oddball task and rhythmic and aperiodic activity during resting sessions with eyes opened and eyes closed. During the post-intervention EEG session, participants also engage in the assigned paced breathing for 5 min (note that this measure was added after seven participants completed their post-intervention EEG visit).

### Trial design {8}

This study is a parallel group, investigator-blind, randomized controlled clinical trial with a 1:1 allocation ratio and a superiority framework. We will examine how daily paced breathing affects plasma Aβ levels and the rate of learning in older adults. Older adults aged between 50 and 70 years old who meet all eligibility criteria are invited to this study. Participants are randomly assigned to one of the two conditions: (1) “slow-paced breathing condition” involving daily memory and attention training (Lumosity brain games) followed by a paced breathing protocol individually adapted to maximize heart rate oscillations or (2) “random-paced breathing condition” involving daily memory and attention training (Lumosity brain games) followed by a paced breathing protocol individually adapted to avoid increasing heart rate oscillations relative to rest. Participants are asked to complete pre- and post-intervention cognitive testing online and engage in 10 weeks of daily brain training and 9 weeks of paced breathing at home. They are also asked to come in for lab visits to provide blood and urine samples to assess Aβ levels; to complete MRI scans to assess PVS volume, hippocampal volume, and LC contrast; to complete neuropsychological tests; and to have brain activity measured during rest and during oddball tasks using EEG.

## Methods: participants, interventions, and outcome

### Study setting {9}

All lab visits are conducted at the University of Southern California (USC). MRI scans are conducted at the USC Dana and David Dornsife Neuroimaging Center (DNI).

### Eligibility criteria {10}

Inclusion criteria are as follows:

- Aged between 50 and 70 years old
- Fluent in English
- Non-pregnant and non-menstruating (for at least the past year)
- Have normal or corrected-to-normal vision and hearing
- Have a home computer with a physical keyboard and have access to reliable Internet
- Agree to provide blood and urine samples
- Healthy adults who weigh at least 110 pounds (for blood draws)
- Agree to devote up to 60 min daily to this study for 12 weeks
- Check an email account regularly
- Have a phone that receives text messages

Exclusion criteria are as follows:

- Have a disorder that would impede performing the breathing intervention (e.g., abnormal cardiac rhythm, heart disease including coronary artery disease, angina, and arrhythmia, cognitive impairment, dyspnea)
- Regularly practicing any relaxation, meditation, or yoga that involves breathing-focused practices lasting for more than an hour each week
- Have regularly played Lumosity games in the past 6 months
- Have participated in heart rate variability biofeedback studies in the USC Emotion & Cognition Lab
- Have any conditions that are not safe for MRI (e.g., metals in the body, claustrophobia, cardiac pacemaker)

### Who will take informed consent? {26a}

Once potential participants pass the online screening, they review the consent form online. The study staff calls potential participants and encourages them to ask any questions they might have about the study. The study staff obtains informed consent, which has been reviewed and approved by the USC Institutional Review Board. Participants digitally sign the consent form.

### Additional consent provisions for collection and use of participant data and biological specimens {26b}

N/A: We do not anticipate the need for additional consent provisions.

## Interventions

### Intervention description {11a}

After an initial week of daily baseline testing at home online, participants undergo 10 weeks of intervention (Table 1). In the first week of intervention (week 2), participants play Lumosity brain games every day followed by measuring their resting heart rate for 5 min with heart rate monitoring via an ear clip. Between weeks 3 and 12, participants do both Lumosity games and breathing practice each day where they engage in ~20–30 min of Lumosity games followed by two sets of 15-min paced breathing practice with heart rate monitoring via an ear clip. They complete the first set of paced breathing practice immediately after Lumosity games and the second set sometime before 11 PM.

**Table 1:** Overview of the study

Timeline	Lab visit + tasks		Home tasks
Week 1	N/A		Home assessments (~45 min/day)
Week 2	Visit 1	EEG, neuropsychological tests	After visit 2: Lumosity games + resting pulse measures (~40 min/day)
	Visit 2	MRI, blood & urine samples, surveys, distribute heart rate monitoring device	
Week 3	N/A		Lumosity games + breathing practice (~60 min/day)
Week 4	N/A		Lumosity games + breathing practice (~60 min/day)
Week 5	N/A		Lumosity games + breathing practice (~60 min/day)
Week 6	N/A		Lumosity games + breathing practice (~60 min/day)
Week 7	Visit 3	MRI, blood & urine samples, surveys	Lumosity games + breathing practice (~60 min/day)
Week 8	N/A		Lumosity games + breathing practice (~60 min/day)
Week 9	N/A		Lumosity games + breathing practice (~60 min/day)
Week 10	N/A		Lumosity games + breathing practice (~60 min/day)
Week 11	N/A		Lumosity games + breathing practice (~60 min/day)
Week 12	Visit 4	EEG, neuropsychological tests	Until visit 5: Lumosity games + breathing practice (~60 min/day) After visit 5: home assessments (~45 min/day)
	Visit 5	MRI, blood & urine samples, surveys, collect heart rate monitoring device	

#### Lab visits

Participants visit the lab five times over the course of the study (Table 1). During week 2 before the intervention begins, participants visit the lab twice to complete baseline measures: once for EEG and neuropsychological tests (visit 1) and once for blood and urine sample collection and MRI assessments (visit 2). During week 7, they visit the lab once to complete blood and urine sample collection and MRI (visit 3). During week 12 after participants have completed the intervention, they visit the lab twice to repeat the same assessments that were completed at baseline (visits 4 and 5).

#### Condition 1: slow-paced breathing

Each day after completing Lumosity cognitive training games, participants are guided to do slow- paced breathing at paces between 10 and 15 seconds per breath. Participants receive continuous biofeedback in the form of a score that is higher the more that their heart rate shows strong oscillatory activity in the 0.04–0.26 Hz frequency range covering normal to slow breathing frequencies. This is described as the ‘“relaxation’” condition to participants. More details are provided in Supplementary information.

#### Condition 2: random-paced breathing

Each day after completing Lumosity cognitive training games, participants are guided to do random-paced breathing at paces between 4 and 6 s per breath. Participants receive continuous biofeedback in the form of a score that is higher the less that their heart rate shows strong oscillatory activity in the 0.04–0.26 Hz frequency range. This is described as the “alertness” condition to participants. More details are provided in Supplementary information.

#### Lumosity brain games

Participants in both conditions play brain games daily. These games are cognitive training games designed to improve various core cognitive skills including memory, attention, processing speed, spatial orientation, mental flexibility, reasoning, and problem-solving skills (https://www.lumosity.com/en/brain-games/). On alternating days, participants are guided by the Lumosity program through 6 out of 12 games shown in Table 2, which take up to ~30 min altogether. On the other days, participants are guided through the other set of six games.

**Table 2:** Description of Lumosity brain games

Set	Game name	Category	Type	Description
1	Familiar faces	Memory	Episodic memory	Participants play a waiter’s role and earn higher tips if they can remember their customers’ names and food orders
1	Tidal treasures	Memory	Episodic memory	On each trial, participants are shown several unique ocean treasures and must choose one that they have not already selected in that round. Rounds can include up to 35 items, and some items are quite similar
1	Lost in migration	Attention	Flanker task	Participants indicate the direction of the central bird in the formation while ignoring the distractors around it
1	Splitting seeds	Attention	Subitizing (visual estimation)	Participants evenly divide a pile of seeds without counting them
1	Pirate passage	Reasoning	Planning	Participants navigate their ship to reach the treasure island without colliding with other pirate ships
1	Ebb and flow	Flexibility	Task switching	Participants view green or orange leaves moving across a pond and indicate the direction of where green leaves are pointing or where orange leaves are moving
2	Memory match	Memory	2-back working memory	Participants must quickly determine whether a flashcard symbol matches the one presented two items ago
2	Word bubble rising	Language	Verbal fluency	Participants generate as many words as possible that start with the same starting letters (e.g., res, medi) within the time limit
2	Raindrops	Math	Arithmetic	Participants perform each math problem inside each raindrop before it reaches the bottom of the screen. Math problems include addition, subtraction, multiplication, and division
2	Penguin pursuit	Attention	Spatial orientation + speed	Participants guide a penguin through a maze to reach a reward of fish at the end before the other penguin does. When the maze rotates, participants must rotate their mental map of the maze and recalibrate the directions to get to the goal
2	Brain shift	Flexibility	Task switching	Participants are shown a letter-number pair (e.g., 5E) on the top or the bottom card. If the letter-number pair shows up on top, participants indicate whether the number is even; if it appears at the bottom, participants indicate whether the letter is a vowel or not
2	Color match 2	Flexibility	Stroop	Participants indicate the color of a written word while ignoring the meaning of the word

### Explanation for the choice of comparators {6b}

An ideal comparison to the slow-paced breathing intervention would be another condition with similar biofeedback information, task demands, and time spent training, but no increases in heart rate oscillatory activity during the training sessions. Thus, we designed a comparison condition where participants do random-paced breathing, which does not increase heart rate oscillations but involves the same type of task as the slow-paced breathing condition.

### Criteria for discontinuing or modifying allocated interventions {11b}

If participants choose to discontinue their participation, they are of course able to do so. Otherwise, once randomized to condition, we do not have any plans to ask participants to discontinue their participation. However, to increase the likelihood that participants successfully complete their daily assigned training sessions during the intervention, if participants miss more than one out of 6 days of home cognitive testing in week 1, they are asked to leave the study before being randomized to a condition.

### Strategies to improve adherence to interventions {11c}

As mentioned above, completion of our initial daily home cognitive testing serves as an initial screen for whether participants have the motivation and time to complete study-related daily tasks. Furthermore, we developed a custom research study application that monitors home practice, sends reminders to practice, and delivers video content at planned intervals that share more details about the study or an encouragement message. Additionally, the application tallies the total earnings to date of each participant from their home practice sessions.

### Plans to promote participant retention and complete follow-up {18b}

The application is also linked to a study dashboard to be accessed only by research team members. The dashboard displays a participant’s status and progress in the study as well as the milestones they have or have not achieved. If participants fall behind on practice for a few days, researchers contact them by email, phone, or video call to check if they have any problems with any aspects of the study, such as device, application, and Internet issues. This dashboard helps researchers coordinate study flow, facilitating intra-team communications. It does not display any information about conditions.

### Relevant concomitant care permitted or prohibited during the trial {11d}

Participants are asked not to play Lumosity games nor to do breathing practice more than the assigned amount. In addition, they are asked not to play other brain games or seek other cognitive/brain applications during their entire participation in the study. If participants play more Lumosity games than what is assigned, they receive an email notification reminding them to adhere to the designated amount. Similarly, if participants exceed the assigned breathing practices, the application restricts them from completing more than the designated amount.

### Provisions for posttrial care {30}

N/A: We do not anticipate the need for provisions concerning posttrial care for this low-risk study.

### Outcomes {12}

#### Primary outcome measures

We will use the false discovery rate methods to correct for multiple comparisons. We will control for false discovery rate at the 0.05 level for three tests of a time-by-condition interaction for three biomarkers (plasma Aβ40, Aβ42, Aβ42/40 ratio).

1. Change in plasma Aβ levels: During the weeks 2, 7, and 12 lab visits, participants provide blood samples to assess Aβ levels. We will compute an aggregate score for plasma Aβ40 and Aβ42 levels after standardizing them using Z scores. This aggregate score will be compared for week 2 (pre-intervention), week 7 (during intervention), and week 12 (post-intervention). We will conduct a time (weeks 2, 7, 12) × condition ANOVA to test for a time × condition interaction in plasma Aβ levels (to assess group differences in change).
2. Change in plasma Aβ42/40 ratio: We will conduct a time (weeks 2, 7, 12) × condition ANOVA to test for a time × condition interaction with plasma Aβ42/40 ratio scores as the dependent variable (to assess group differences in change).

#### Secondary outcome measures

1. Change in brain PVS volume: We will use T1w and T2w MRI protocols from the Human Connectome Project [33] to acquire MRI data. We will employ the image processing pipeline based on Frangi filter 3D vesselness tubular structure probability estimation [34] to estimate PVS volume in centrum semiovale white matter at each assessment point. PVS volume is measured during weeks 2, 7, and 12. We will test whether there are group differences in change in PVS volume.
2. Change in hippocampal volume: We will use a high-resolution hippocampal slab sequence [35]. It is oriented perpendicular to the long axis of the hippocampus, which allows a high in-place resolution for segmentation and sufficient SNR (due to the thick slices). Hippocampal volume is measured for weeks 2, 7, and 12. We will test whether there are group differences in change in hippocampal volume.
3. Brain training performance on 12 Lumosity games during the breathing intervention (controlling for brain training performance pre-intervention): We will compute a general learning factor across performance on all 12 games played daily during the intervention period (weeks 3–11). We will also assess game performance in the pre-intervention week 2 to provide a baseline performance measure for each participant and statistically account for it.

##### Other pre-specified outcome measures

1. Change in plasma pTau-181/tau ratio: We will conduct a time (weeks 2, 7, 12) × condition ANOVA to test for a time × condition interaction with plasma pTau-181/tau ratio as the dependent variable (to assess group differences in change).
2. Change in urine Aβ42: We will conduct a time (weeks 2, 7, 12) × condition ANOVA to test for a time × condition interaction with urine Aβ42 as the dependent variable (to assess group differences in change).

##### Exploratory outcome measures

1. Changes in hippocampal subregion volumes
2. Comparisons of PVS volume changes across different brain areas
3. Changes in LC MRI contrast
4. Changes in cognitive function measured by various cognitive assessments listed in Table 4
5. Changes in emotional memory and well-being measured by various emotion-related assessments listed in Table 4
6. Changes in EEG activity during the active and passive oddball tasks
7. Changes in EEG activity during rest
8. Changes in heart rate variability (HRV) during rest

### Participant timeline {13}

Table 3 shows the participant timeline.

**Table 3:**
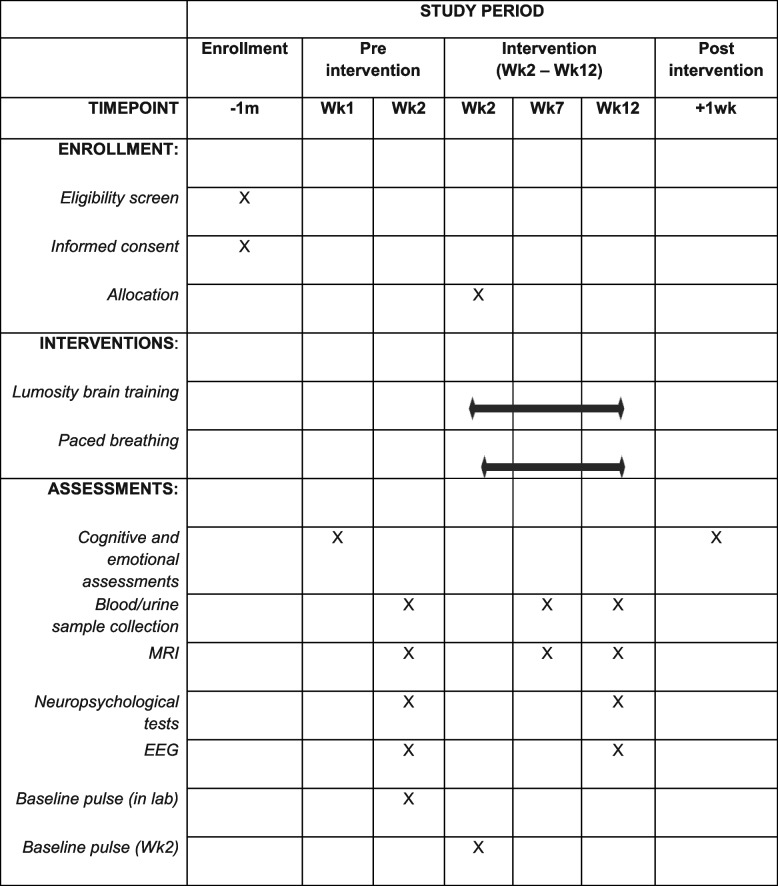
Participant timeline

### Sample size {14}

We conducted a preliminary study to determine the reliability of the PVS volume measure using the Dornsife 3T scanner, scanning five participants each on three separate weeks at the same time of day. Whole-brain estimates of PVS volume were highly reliable (intraclass correlation, ICC_(3,k)_ = 0.969, *F*(4.20) = 32.12, *p* < .001). This high measurement reliability provides excellent power to detect the within (time point: week 2, week 7, week 12) × between (paced breathing intervention: slow, random) interaction we will be testing. In fact, with 0.95 correlation among repeated measures, at *α* = .05, G*Power estimates that we will have 95% power to detect small (*f* = 0.10) effects in a repeated-measures ANOVA with just *N* = 14 in each condition (total *N* = 28). Thus, we are well powered to detect changes in PVS volume.

For the AD biomarkers, we used correlations between pre- and post-intervention time points in our previous clinical trial [36] to estimate reliability. We estimated effect sizes based on the pre-post difference within the older adult increase-oscillations group that had done slow-paced breathing. Using the most conservative parameters based on these estimates (the lowest reliability was 0.71, and the lowest effect size was a medium one), we would need only *N* = 13 in each condition (total *N* = 26) to have 95% power to detect effects at *α* = .05. However, we do not have estimates of reliability or effect sizes for additional exploratory assays we will conduct. Thus, we have powered our study to be able to detect small-to-medium effect sizes (*f* = 0.2) with 0.7 correlation among repeated measures with 95% power at *α* = .05. With these parameters, we need a total N of 42 with three assessments each (weeks 2, 7, 12).

Given these power analyses, we aim for a final completed N of at least 48 for our analyses. To achieve this objective, we are enrolling more than 48 participants to accommodate for missing data due to dropouts and potential individual session data quality issues, with exact enrollment N depending on dropout rate.

### Recruitment {15}

Participants are recruited from the greater Los Angeles community. We use emails, letters, flyers, online ads/postings (e.g., our lab’s Healthy Minds volunteer pool), and personal solicitation (e.g., at senior center presentations). We also work with a recruitment company called Trialfacts to advertise the study and recruit participants.

## Assignment of interventions: allocation

### Sequence generation {16a} and concealment mechanism {16b}

Our custom application applies a blocked randomization scheme (stratified by sex at birth with block size of 2) and assigns participants to one of the two conditions when they receive a lab-issued laptop for completing the intervention after MRI assessments in visit 2. The application asks participants their sex and then counts the number of existing participants who are (a) already assigned to condition and (b) of the same sex as the incoming participant. If that number is even (0 is even), then the application randomly assigns the incoming participant to condition. Otherwise, it finds the condition of the most recently assigned participant of that sex and gives the incoming participant the opposite condition.

This assignment to condition is not shown to the researchers or the participant and does not determine any different activity in the application until participants have completed 6 days of “baseline” Lumosity game plays. Thus, the first time the application does anything different based on condition is about a week after visit 2, when the participant is at home. This procedure maintains researchers’ blinding to condition.

We selected the block size of two as it will best balance the N’s in the two conditions. Although this block size means that the condition of the second participant in each block can be predicted by the preceding participant in that block, this predictability is not an issue for our study because our study team responsible for recruiting and scheduling participants are blinded to randomization outcomes and do not conduct the randomization process. The condition of the first participant in each block is randomized, so this is not an alternating sequence. Thus, even if schedulers were not blinded, due to the long delay between enrollment and randomization, they would not be able to sequence scheduling to preferentially assign participants to a condition. Session scheduling occurs at least 2 weeks before actual randomization and 3 weeks before the application provides any differential training based on condition. Additionally, we enroll 2–3 new participants on average each week, meaning that when each participant is enrolled, there are other enrolled participants queued up to be randomized before them.

### Implementation {16c}

The application generates the allocation sequence and assigns participants to intervention conditions. Three research staff members enroll participants.

## Assignment of interventions: blinding

### Who will be blinded {17a}

All investigators interacting with participants during in-lab sessions are blinded to the intervention assignments. This is possible since the application randomly assigns participants to interventions which will not be revealed until they start the first intervention session at home and because both interventions involve pulse monitoring during paced breathing, allowing investigators to troubleshoot any issues with the pulse monitoring device with participants in the same way regardless of condition.

However, just in case an investigator develops some idea about which condition a particular participant is in from talking with them during the course of the study, we have implemented a second layer of blinding for analysts for the primary and secondary outcomes. Using a scripting process coordinated by our software programmer and database manager, who has no contact with participants and will not conduct any analyses, each analyst will be provided with the set of raw data that they intend to analyze with each participant’s data identified with a new ID. Thus, analysts will not be able to use someone’s study ID to identify who they were. Furthermore, the two conditions will be indicated with two letters (e.g., “H,” “X”) that are randomly selected separately for each data subset (e.g., structural scans for MRI PVS and hippocampus volume, plasma biomarkers, Lumosity data). Analysts will use these two anonymized labels to compare intervention conditions. Analyses will be conducted independently for each of the data types associated with our primary and secondary outcomes. Thus, in any team meetings discussing analyses before unblinding, researchers will not be able to use any differential condition outcomes for one type of data to predict which condition is which for another type of data. Our database manager/study software programmer will manage this blinding process and store the condition label assignment associated with each data type to provide unblinding when analyses are complete, as well as the mapping from the analysis-specific participant number to the study-wide participant number.

### Procedure for unblinding if needed {17b}

N/A: Unblinding during study of blinded research staff is not needed.

## Data collection and management

### Plans for assessment and collection of outcomes {18a}

Data are collected at participants’ home and in the lab (Table 4). Each day in the first and last weeks of the study (i.e., weeks 1 and 12), participants complete cognitive tests, emotion questionnaires, and other surveys at home. During their lab visits, participants provide blood and urine samples, undergo MRI scans, and complete EEG assessments, neuropsychological tests, and other surveys.

**Table 4:** Description of assessments

Category	Type	Assessment	Time of data collection (week)	Location of data collection	Data storage during data collection
Home assessments (cognitive tests)	Verbal learning and memory	California Verbal Learning Test [37]	1, 12	Home	Amazon’s cloud storage system
	Working memory	N-back task [38]			
	Episodic memory	Face-name associative memory task [39]			
	Episodic memory	Pattern separation task [40]			
	Executive function	Task-switching task [41, 42]			
	Executive function	Flanker task [43]			
	Visuospatial function	Spatial orientation task [44]			
	Verbal fluency	Thurstone Word Fluency Test [45]			
Home assessments (emotion questionnaires)	Emotional memory	Emotional memory task [46]			
	Emotional memory	Mood prediction and memory			
	Theory of mind	Reading the mind in the eyes test [47]			
	Mood	Positive and Negative Affect Schedule (PANAS) [48]			
	Stress	Daily Inventory of Stress Events [49]			
	Depression/anxiety/stress	Depression Anxiety Stress Scales-Short Form (DASS-21) [50]			
	Mindfulness	Five Facet Mindfulness Questionnaire (FFMQ) [51, 52]			
Home assessments (other questionnaires)	Sleep	Epworth Sleepiness Scale [53]			
	Sleep	Karolinska Sleepiness Scale [54]			
	Demographics	Demographic information	1		
	Physical activity	Physical activity ratings [55]			
	Survey	Post study survey	12		
Blood sample	N/A	Plasma amyloid beta levels	2, 7, 12	Lab	Lab freezer
		Plasma pTau			
		Plasma total tau			
Urine sample	N/A	Amyloid beta levels			
MRI session	Structural MRI	T1-weighted sequence	2, 7, 12	Imaging center	Flywheel
		T2-weighted sequence			
		LC low resolution (proton density-weighted sequence)			
		LC high resolution (proton density-weighted sequence)			
		Hippocampal high resolution (T2-weighted sequence)			
		Fluid-attenuated inversion recovery (FLAIR)	12		
	Pulse	Pulse oximeter recording photoplethysmogram (PPG) signal from finger	2, 7, 12		Lab laptop and external hard drive
EEG	N/A	Oddball task (passive and active) [56, 57]	2, 12	Lab	Lab computer and flywheel
		Resting state EEG measure (with eyes open and closed)			
		Training mimicking EEG recording	12		
Neuropsychological tests	Cognition	Trials A & B [58]	2, 12		Amazon’s cloud storage system
		Animal fluency [59]			
		Multilingual naming test [60]	2		
		Montreal Cognitive Assessment (MoCA) [61]			
		Functional Activities Questionnaire [62]			
Surveys	N/A	Profile of Mood States (POMS) [63]	2, 7, 12		Lab computer and flywheel
		Daily Inventory of Stressful Events (DISE) [49]			
		Sleep survey			
Baseline pulse measures	Pulse during rest and during 4-s paced breathing	Pulse oximeter recording photoplethysmogram (PPG) signal from earlobe	2	Lab after MRI	Amazon’s cloud storage system
	Pulse during rest			Home	
Breathing training	Pulse during paced breathing		3–11		
Cognitive training	Brain games	Brain game performance	2–11		

Participants’ Lumosity game performance is collected at home and saved in the Lumosity database in a securely de-identified format. Lumosity emails game reports to a dedicated account for this research project every day. These are automatically processed, and the raw reports are saved in Amazon S3, while selected data from the reports are extracted and stored in Amazon DynamoDB. Pulse data during breathing practice are also collected at home, which are stored in Amazon’s cloud storage system.

Before enrolling participants, we pilot tested all aspects of data collection to ensure data quality. Researchers also monitor incoming data. For instance, during the MRI sessions, researchers operating the scanner inspect the T1 and T2 scans, and if excessive motion is detected (e.g., severe ringing and blurring artifacts), rerun these scans. Also, if there is sufficient time, duplicate runs of the LC-targeted scans are run to increase measurement reliability.

### Data management {19}

After data collection has been completed, all data will be stored on flywheel (https://uscdni.flywheel.io). At the conclusion of the study, data in de-identified form will be shared using OpenNeuro and Open Science Framework, which are free and open platforms for sharing and archiving scientific data. All data will be kept indefinitely in electronic form and may be presented in aggregate form at conferences or in journal articles.

### Confidentiality {27}

Screening is conducted online. Screening responses from potential participants are used to determine eligibility but are not stored for each participant and so are not tied to an individual. We store the number of times the screening is passed or failed. For all participants, we retain contact information (i.e., email and phone) and sex for the duration of the study. Additionally, for ineligible participants, the reasons for ineligibility (i.e., did not meet inclusion criteria, declined to participate) are retained.

For enrolled participants, we track daily completion of the home assessments and interventions and email and/or text them reminders to complete them. In order to perform this, their emails are stored as part of their password-protected user accounts. Those staff researchers involved in recruitment and monitoring participants’ completion of home practice sessions have access to participants’ personal information (email, phone numbers).

### Plans for collection, laboratory evaluation, and storage of biological specimens for genetic or molecular analysis in this trial/future use {33}

Blood and urine samples are kept in −80 °C freezers with de-identified codes until they will be shipped in one batch to be assayed.

## Statistical methods

### Statistical methods for primary and secondary outcomes {20a}

Data will be analyzed using the SPSS 28 software and R package. The data normality will be evaluated through the Shapiro-Wilk test. If the data are not normally distributed, transformation will be carried out to make the data follow a normal distribution.

As described in the outcomes section, most of the outcome measures (plasma Aβ levels, plasma Aβ42/40 ratio, PVS volume, pTau181/tTau, urine Aβ42) will each be analyzed using a time × condition ANOVA. Hippocampal volume (secondary outcome measures) will be analyzed using a time × condition ANCOVA controlling for intracranial volume estimate. If, for any of these outcomes, the measurements at week 2 (pre-intervention) show a significant difference across the conditions, we will instead conduct an analysis of covariance (ANCOVA) on week 7 and week 12, respectively, controlling for week 2 data (and controlling for intercranial volume in the case of hippocampal volume).

Brain training performance on 12 Lumosity games during the breathing intervention will be analyzed using multivariate statistics (e.g., task partial least-squares correlation [64]) to estimate latent task-independent learning trajectories [65]. Learning curves will then be compared across experimental groups, and associations with variables of interest will be determined.

### Methods in analysis to handle protocol nonadherence and any statistical methods to handle missing data {20c}

We will follow the gold standard for clinical trials of “intention-to-treat,” which means there is no minimum amount of intervention that someone needs to complete. This method allows the investigator to draw unbiased conclusions regarding the effectiveness of an intervention on outcome variables [66].

The missing data patterns for the primary and secondary outcomes (i.e., plasma and MRI data) and reasons for missingness will be summarized. For criteria with less than 5% missing data, we will not perform a missing data imputation method and delete the cases with missing data. For criteria with more than 5% missing data, we will categorize the nature of these missing data—whether they are completely randomly missing, randomly missing, or non-randomly missing [67]. If data missing at random is assumed, we will perform sequential multiple imputations by chained equations (MICE) implemented in the mice R package with 20 imputations [68]. Missing values will be imputed separately by the two conditions [69]. If one condition consistently has more missing values than the other significantly, it might indicate data is not missing at random, and sensitivity analysis will be used [67].

In terms of the analysis of brain training performance on 12 Lumosity games, the time series of game-play performance will be downsampled to accommodate potentially missing values [65]. If after downsampling no performance data are available for complete temporal bins, these entries will be imputed from neighboring bins using linear interpolation.

### Methods for additional analyses (e.g., subgroup analyses) {20b} and interim analyses {21b}

N/A: Currently, there are no interim and subgroup analyses planned.

### Plans to give access to the full protocol, participant level-data, and statistical code {31c}

The full protocol will be shared on ClinicalTrials.gov. See the “Data management” section for public data sharing.

## Oversight and monitoring

### Composition of the coordinating center and trial steering committee {5d} and composition of the data monitoring committee and its role and reporting structure {21a}

Based on National Institutes on Aging (NIA) guidelines, our study is not in the category of studies that would require a Data and Safety Monitoring Board (DSMB), as it is not a phase III clinical trial, does not involve multiple field sites, and the experimental manipulations do not involve high risk of adverse events. The primary manipulation involves paced breathing, and risks are minimized via our exclusion criteria. According to the US Department of Health & Human Services (HHS), 45 CFR 46, a study involving more than minimal risk, is one in which the probability and magnitude of harm or discomfort anticipated in the research are greater than those ordinarily encountered in daily life or during the performance of routine physical or psychological examinations or tests. The USC IRB has approved a protocol for our study and agreed that it qualified for expedited review due to its minimal risk.

Our data safety monitoring team comprises Drs. Mather (PI) and Nashiro (co-I), with the PI taking responsibility for reporting any adverse events immediately to the IRB. We monitor participant safety and well-being by regularly asking participants to fill out questionnaires to probe them as to whether they have experienced any adverse outcomes from the blood draws. We also monitor for any adverse events during MRI and EEG sessions. If there is a serious adverse event that requires medical attention during a lab visit, we will call 911.

### Adverse event reporting and harms {22}

Adverse events are categorized by the data safety monitoring team based on their attribution (related or unrelated) and severity. The clinical trial involves paced breathing in both conditions, and all other procedures/activities are the same across conditions so we do not anticipate unmasking will be necessary to determine attribution. Potential study-related adverse events may occur from the blood draw; to track these, we include a questionnaire that participants are asked to complete at home (using an online survey tool called Qualtrics) a few days after each of the three blood draws to ask them how painful the blood draw was compared to typical blood draws, if they experienced any bruising, and if they felt light-headed or faint afterwards (unlikely given the small volume of blood being drawn) or any other adverse events. Qualtrics automatically alerts the investigators if the individual indicates any adverse events in response to the blood draw questionnaire. Any of these individual responses to the questionnaires indicating adverse events beyond the level of mild lightheadedness or mild blood draw pain/bruising are reported to the IRB, and the study coordinator contacts the participant to check in on how they are feeling and if they have any new symptoms. If a participant loses consciousness during blood draw or as a result of the breathing practice, the participant will be precluded from continuing with any further blood draws/breathing practices. In most cases, this will preclude further participation in the study, but if the study can be completed without repeating the precipitating event (e.g., losing consciousness occurred at the second blood draw, and so no more blood draws will occur), they will be allowed to continue. All phlebotomists in this study are certified in basic life support. Any adverse events occurring during the MRI and EEG sessions are also reported to the IRB.

### Frequency and plans for auditing trial conduct {23}

N/A: Currently, no audits are planned.

### Plans for communicating important protocol amendments to relevant parties (e.g., trial participants, ethical committees) {25}

If protocol modifications were made (e.g., changes to eligibility criteria, outcomes, analyses), we plan to inform all investigators, submit an amendment to the IRB, and make changes in ClinicalTrials.gov.

### Dissemination plans {31a}

As required, we have registered the trial at the clinical trials government website prior to enrolling the first subject (see Clinicaltrials.gov NCT05602220). During the course of the study and afterwards, we confirm accuracy of record content, resolve problems, and maintain records including content update and modifications. We will also be responsible for results reporting and adverse events reporting at the conclusion of the project. We plan to publish the results in peer-reviewed journals.

In general, we follow USC clinical trial regulations and procedures. For instance, informed consent documents for the clinical trial(s) include a specific statement relating to posting of clinical trial information at ClinicalTrials.gov. As required, the recipient institution (USC) has an internal policy in place to ensure that clinical trials registration and results reporting occur in compliance with policy requirements.

## Discussion

This trial aims to reproduce our initial findings that daily slow-paced breathing reduces overall plasma Aβ levels (Aβ40 and Aβ42) and increases the plasma Aβ42/Aβ40 ratio. Our secondary objectives are to test whether daily slow-paced breathing will affect PVS volume, hippocampal volume, and the rate of learning. We predict that the intervention will have positive effects on brain structures, reflecting brain clearance of Aβ. If intervention-induced enhanced brain clearance processes reduce Aβ aggregation and thereby restore some synaptic plasticity, we should be able to detect improvement in the rate of learning.

To date, we lack evidence from randomized trials that low risk and inexpensive interventions improve clearance of brain metabolic waste products, a critical function to prevent the accumulation and aggregation of Aβ peptides and tau proteins. This gap is reflected in the lack of success of behavioral interventions (including sleep, diet, and exercise) in improving either positron emission tomography (PET) measures of brain amyloid burden, CSF Aβ, or plasma Aβ42/40 ratios. Despite evidence that acute manipulations such as sleep deprivation [70] and stress [71] can exacerbate Aβ biomarker profiles, the field has yet to identify behavioral interventions that improve Aβ biomarker profiles in the brain or periphery. Slow-paced breathing is a low risk and easily adopted intervention and so could be of wide use if it does indeed help slow some aspects of the AD pathological process.

### Trial status

The study recruitment started on 12 January 2023, and data collection is anticipated to be completed by 30 November 2023. The trial is aligned with IRB protocol version 11, approved on 15 May 2023.

### Supplementary Information

**Additional file 1:** **Supplementary Table 1.** Methodological differences between the HRV-ER and the HeartBEAM clinical trial. **Supplementary Table 2.** Sequence of events in the two conditions. **Supplementary Table 3.** Selection of regimes in the resonance frequency condition depending on how many regimes’ coherence values (from previous practice sessions) overlap with the regime that currently has the highest average coherence value from previous practice sessions. **Supplementary Table 4.** Selection of regimes in the random pace condition depending on how many regimes’ coherence values (from previous practice sessions) overlap with the rest sessions’ coherence.

## Abbreviations

Aβ: Amyloid beta
AD: Alzheimer’s disease
DNI: Dornsife Neuroimaging Center
DSMB: Data and Safety Monitoring Board
EEG: Electroencephalography
HHS: Health & Human Services
LC: Locus coeruleus
MoCA: Montreal Cognitive Assessment
MRI: Magnetic resonance imaging
NIA: National Institutes on Aging
NIH: National Institutes of Health
PVS: Perivascular space
USC: University of Southern California
