# Supplementary material for: Heart rate and breathing effects on attention and memory (HeartBEAM): study protocol for a randomized controlled trial in older adults
Source: Trials. 2024 Mar 15;25:190. doi: 10.1186/s13063-024-07943-y (PMC10941428; doi:10.1186/s13063-024-07943-y)
Supplement: Supplementary file 1 — Additional file 1: Supplementary Table 1. Methodological differences between the HRV-ER and the HeartBEAM clinical trial. Supplementary Table 2. Sequence of events in the two conditions. Supplementary Table 3. Selection of regimes in the resonance frequency condition depending on how many regimes’ coherence values (from previous practice sessions) overlap with the regime that currently has the highest average coherence value from previous practice sessions. Supplementary Table 4. Selection of regimes in the random pace condition depending on how many regimes’ coherence values (from previous practice sessions) overlap with the rest sessions’ coherence. [file 13063_2024_7943_MOESM1_ESM.docx]

**Supplementary Information**

Supplementary Table 1. Methodological differences between the HRV-ER and the HeartBEAM clinical trial.

| **Study component** | **HRV-ER clinical trial [1]** | **HeartBEAM clinical trial** |
| --- | --- | --- |
| Intervention period | 5 weeks | 9 weeks |
| Experimental condition | slow paced breathing | slow paced breathing + Lumosity games |
| Control condition | using personalized strategies to minimize heart rate oscillations | random paced breathing to avoid increasing heart rate oscillations (more details below) + Lumosity games |
| Feedback during intervention | show the on-going history of heart rate fluctuations | display a biofeedback summary score (more details below) |
| Blood sample collection | pre and post intervention (4 weeks apart) | pre (a week before starting intervention), midpoint (4 weeks after starting intervention) and post intervention (after 5 weeks more weeks of intervention) |
| Urine sample collection | N/A | pre (a week before starting intervention), midpoint and post intervention (5 weeks between timepoints) |
| Cognitive tasks | NIH Toolbox Cognition Battery | Cognitive tests described in Table 4 in the main text |
| Structural MRI | T1, LC low resolution | T1, T2, LC low resolution, LC high resolution, hippocampal high resolution scans (Table 4) |

HRV-ER = heart rate variability biofeedback and emotion regulation

HeartBEAM = heart rate and breathing effects on attention and memory

LC = locus coeruleus

*Overview of the study software application*

Participants will sign up for the application with their email and the password they created for the online baseline cognitive tasks/questionnaires. From that account, the application will have access to their first name, email, phone number, gender (for stratifying randomization) and year of birth (for letting Lumosity know which age decade they fall in, e.g., 50’s or 60’s). Throughout the study, participants will be able to see how many training sessions they have completed, how much money they have earned, and earn medals/badges for completed training. For each training session, participants will receive instructions related to their training. They will also receive feedback on how well they are performing. Participants will receive reminders when they fail to complete training sessions.

After the participant has signed up for the application, the application will (1) randomly assign participants to one of two conditions, Slow Paced Breathing (“Slow Paced”) or Random Paced Breathing (“Random Paced”), and (2) randomly assign participants a Lumosity username and password. Throughout the rest of the intervention, the application will repeat the most effective training paces based on the average coherence score (reverse scored for Random participants) for that particular pace. It will also monitor training, send reminders to train, and will deliver video content at planned intervals that share more details about biofeedback training, the study, or an encouragement message. Additionally, the application will tally the total earnings to date of each participant from their home training sessions.

Finally, a study dashboard only to be accessed by research team members will display a participant’s status and progress in the study as well as the milestones they have or have not completed. This dashboard will serve to automate study flow, facilitating intra-team communications.

# *Definitions*

## Cadence

A regime that includes breath holds has a cadence of 1:1:1 for inhale, hold and exhale phases. A regime without breath holds has a cadence of 1:1 for inhale and exhale phases. These are the only two cadences used in the study.

## Coherence

“Coherence” is a number obtained from the HeartMath emWave application [2] that is a measure of heart rate variability. It is calculated by computing the peak spectral frequency power within the frequency range 0.04 - 0.26 Hz and determining the ratio of peak power to total power in the 0.0 - 0.4 Hz range minus peak power. We will be providing participants in the random condition with feedback designed to keep their coherence score close to their resting state coherence score measured before the start of the breathing intervention, while providing participants in the slow paced condition with feedback designed to increase it.

## Pace

A certain number of breaths per minute that a participant should try to breathe at. Sometimes also used to mean a particular breathing regime.

Paced Breathing

Breathing done at the pace specified by a given regime. The participant will get a visual display showing them when to inhale, when to exhale and (if specified) when to pause their breathing.

## Regime

A regime specifies a particular pattern of breathing that a user should follow. It is defined by four characteristics: total duration (typically five minutes), pace (a certain number of breaths per minute), a “hold position” (whether a user should pause after inhalation, after exhalation or not at all), and whether or not the breathing pace should be randomized. A randomized regime varies the length of each inhalation, exhalation, and hold (if specified) but does so in a way that over the course of the total duration the average number of breaths per minute matches the pace specified for the regime. In non-randomized regimes, all inhalations and exhalations (and holds, if specified) are the same length.

In a randomized regime without a hold, the length of each inhalation or exhalation should be in the range (60 / regime pace) +/- 1 seconds. Any inhalations/exhalations that are of different lengths should differ by at least 50ms. In a randomized regime with a hold, the length of each inhalation, exhalation and hold should be in the range (60 / regime pace) +/- 0.66 seconds. Any inhalations/exhalations/holds that are of different lengths should differ by at least 33ms.

If the regime describes a non-whole number of breaths then the total number of breaths will be increased to the next whole number. Randomization can also result in a segment being slightly longer or shorter than specified in the regime. (It may also result in a slightly different number of breaths per minute, though over a long enough duration that is less likely to happen.)

Regimes always begin at the start of an inhalation and end either at the end of an exhalation or the end of a post-exhalation hold.

## Rest Breathing

Breathing done at no particular pace while the participant sits quietly. Participants receive no visual display or biofeedback during this type of breathing.

##

## Segment

A time during which the participant breathes following the specification of a single regime. This will typically be close to five minutes.

##

## Session

A series of consecutive segments without any pauses, breaks or interruptions between them. The goal is for each session to consist of three segments and for participants to do two sessions each day. Sessions should never be longer than three segments, but may be shorter if a participant interrupts one, their computer crashes, etc.

## *Detailed Overview*

### Week 2: Setup in the lab

The setup will happen in the lab when the participant receives his or her computer. It consists of:

- Logging in to the home training app (using the same email address and password the participant used for the cognitive baseline portion of the study)
- Entering information needed to assign them to condition (and being assigned to condition, though they don’t know this)
- Doing 5 minutes of rest breathing
- Doing 5 minutes of paced breathing at 15 breaths per minute, no holds, non-randomized.

### Week 2: Lumosity + Rest Breathing at home

Week 2 begins when the participant first uses the app at home. Participants cannot do Lumosity more than once per day. (This is controlled by Lumosity.)

In Week 2, participants will do approximately 15 minutes of Lumosity games followed immediately by 5 minutes of rest breathing each day.

Week 2 ends when both of the following are true:

- The participant has completed at least 2 gameplays of all 12 Lumosity games used in this study and 31 total gameplays across all games/days.

*Explanation*: For participants who play 6 games per day as instructed, they would not meet these criteria until six days of game play. If participants miss 1-5 game plays across days (e.g., due to quitting early), when doing our statistical modeling at the end of the study we will impute the values for missing data based on predicted outcomes from other participants and their own learning trajectory. Allowing some missing game plays helps to avoid holding up a participant who is missing one game play in Week 2 for two extra days until they finally have played that particular game again.

### Week 3: Lumosity + Paced Breathing at home

In Week 3 participants will do two sessions per day. The first session will consist of approximately 20-30 minutes of Lumosity games followed immediately by 15 minutes of paced breathing. Later that day (or immediately afterwards if they choose) they will do a second session of just 15 minutes of paced breathing. Week 3 ends when the participant comes into the lab for their final MRI and EEG sessions and returns the study laptop.

#### Regime Selection

Participants will train under a variety of regimes through the course of the study, and those will differ depending on which condition the participant is assigned to. Because regimes don’t come into play until Week 3, “Day 1” below refers to the first day of Week 3. Note that while we hope days will be consecutive, it’s always possible that a participant will skip training on any given day, so “Day N” refers to the Nth day that a participant trains after starting Week 3, not the Nth calendar day after Week 3 begins.

At the start of the day the application will generate the set of possible regimes (see below) and randomize the order. Regimes will then be delivered in that order for that day. All regimes are five minutes long. All days have six regimes (split across two sessions), though in many cases only three are described below. In those cases, we generate a list of six regimes by using each of the three twice.

Supplementary Table 2. Sequence of events in the two conditions.

##### Day 1

| Slow Paced Condition | Random Paced Condition |
| --- | --- |
| 4, 5 or 6 breaths per minute, no holds, no randomization | 10, 12 or 15 breaths per minute, no holds, with randomization |

##### Day 2

| Slow Paced Condition | Random Paced Condition |
| --- | --- |
| 4, 5 or 6 breaths per minute, with post-inhalation holds, no randomization | 10, 12 or 15 breaths per minute, with post-inhalation holds, with randomization |

##### Days 3 and 4

| Slow Paced Condition | Random Paced Condition |
| --- | --- |
| 3 regimes, with post-inhalation holds and without randomization, of 4, 5 and 6 breaths per minute.  3 regimes, without holds and without randomization, of 4, 5 and 6 breaths per minute. | 3 regimes, with post-inhalation holds and with randomization, of 10, 12, and 15 breaths per minute.  3 regimes, without holds and with randomization, of 10, 12, and 15 breaths per minute. |

##### Subsequent Days

Regimes are algorithmically selected as described below.

Slow Paced Condition

For each regime (typically five minutes), calculate the average coherence and the 90% confidence interval around that mean (using all of the segments the participant has done under that regime, but only the last four minutes of each segment). Find the regime with the highest average coherence. Find all of the regimes that either (a) lack a confidence interval (which will be those with <2 segments of data) or (b) whose 90% confidence interval overlaps with that highest average coherence value. Given the number of overlapping regimes, do the following:

Supplementary Table 3. Selection of regimes in the resonance frequency condition depending on how many regimes’ coherence values (from previous practice sessions) overlap with the regime that currently has the highest average coherence value from previous practice sessions.

| Number of regimes overlapping with the best regime (including the best regime - it overlaps with itself) in their coherence values | Outcome |
| --- | --- |
| 1 | If this is the first time that this regime is the single best regime, create two new regimes: one that is a ½ breath per minute below the pace of this regime and one a ½ breath per minute above. Both of the new regimes should have the same cadence and randomization as this regime. The best regime and the two new regimes should each be used twice, in a random order.  If this is the second time this regime is the single best regime, follow the above procedure but use +/- ¼ breath per minute as the differential for the new regimes. For the third time use +/- ⅛ breath per minute, etc. |
| 2 | Pick a random number between 0 and 1. If it is < 0.5, fill the first 5 slots with the best regime. If it is >= 0.5, fill the last 5 slots with the best regime. Fill the remaining slots with the other regime. |
| 3 | Pick a random number between 0 and 1. If it is < 0.5, fill the first 4 slots with the best regime. If it is >= 0.5, fill the last 4 slots with the best regime. Fill the remaining slots with the remaining potential regimes, ordered randomly. |
| 4-5 | Pick a random number between 0 and 1. If it is < 0.5, fill the first 2-3 slots with the best regime. If it is >= 0.5, fill the last 2-3 slots with the best regime. Fill the remaining slots with the remaining potential regimes, ordered randomly. |
| 6 | Use each regime once, in random order. |
| 6+ | Randomly select six, without replacement, and use each of these once, in a random order. |

Random Paced Condition

In the random paced condition we want to select a regime whose average coherence is no higher than the average coherence the participant achieves during rest breathing. To accomplish this we do the following:

Compute the mean rest breathing coherence using the Week 2 sessions, using only the last four minutes of each session. Next, compute the mean coherence and 90% confidence interval around that coherence for each Week 3 regime, using only the last four minutes of each session. Now find all regimes that either (a) lack a confidence interval (which will be those with <2 segments of data) or (b) whose 90% confidence interval overlaps with the mean rest breathing coherence. Given the number of coherence-overlapping regimes, do the following:

Supplementary Table 4. Selection of regimes in the random pace condition depending on how many regimes’ coherence values (from previous practice sessions) overlap with the rest sessions’ coherence.

| Number of regimes overlapping in coherence with rest sessions | Outcome |
| --- | --- |
| 0 | Use the regime with the average coherence that is closest to mean rest breathing coherence for all segments for the day. |
| 1 | Use this regime for all segments for the day. |
| 2 | Use each regime three times (randomize the order of the six) |
| 3 | Use each regime twice (randomize the order of the six) |
| 4-5 | Use each regime once, then randomly select enough repeats (without replacement - that is, if you randomly pick regime A for the first, don’t pick it again for the second) to give you six regimes. (Then randomize the order of the six.) |
| 6 | Use all of the regimes |

**References**

1. Yoo HJ, Nashiro K, Min J, Cho C, Mercer N, Bachman SL, et al. Multimodal neuroimaging data from a 5-week heart rate variability biofeedback randomized clinical trial. Scientific Data. 2023;10(1):503.

2. Heartmath. EmWave Pro Plus. 2016.
